# The Aging Features of Thyrotoxicosis Mice: Malnutrition, Immunosenescence and Lipotoxicity

**DOI:** 10.3389/fimmu.2022.864929

**Published:** 2022-06-02

**Authors:** Qin Feng, Wenkai Xia, Guoxin Dai, Jingang Lv, Jian Yang, Deshan Liu, Guimin Zhang

**Affiliations:** ^1^Center for Pharmacological Research, State Key Laboratory of Generic Manufacture Technology of Chinese Traditional Medicine, Lunan Pharmaceutical Group Co., Ltd., Linyi, China; ^2^Department of Traditional Chinese Medicine, Qilu Hospital of Shandong University, Jinan, China

**Keywords:** aging, thyrotoxicosis, malnutrition, immunosenescence, lipotoxicity, proteomics, metabolomics

## Abstract

The problem of aging is mainly the increase of age-related diseases, and elderly patients have longer hospitalization and worse prognosis. Poorer nutritional status and immunosenescence may be predisposing and severe factors. The mechanism of the high incidence of diseases and poor prognosis behind aging is complex. Finding suitable aging models is of great significance to find strategies to prevent aging related events. In this study, the relationship between thyrotoxicosis and aging was investigated in mice. The results of routine blood tests and flow cytometry showed that immunosenescence occurred in thyrotoxicosis mice, which was characterized by a significant decrease in neutrophils, lymphocytes, CD4+/CD8+ and CD4+IFN-γ+ lymphocytes. Biochemical examination results showed that there were hypocholesterolemia, hypolipoproteinemia, and hyperlipidemia in thyrotoxicosis mice. Serum proteomics analysis showed that the downregulation of complement and coagulation proteins was another manifestation of declined immunity. Moreover, proteomics analysis showed that many downregulated proteins were related to homeostasis, mainly transport proteins. Their downregulation led to the disturbance of osmotic pressure, ion homeostasis, vitamin utilization, lipid transport, hyaluronic acid processing, and pH maintenance. Serum metabolomics analysis provided more detailed evidence of homeostasis disturbance, especially lipid metabolism disorder, including the downregulation of cholesterol, vitamin D, bile acids, docosanoids, and the upregulation of glucocorticoids, triglycerides, sphingolipids, and free fatty acids. The upregulated lipid metabolites were related to lipotoxicity, which might be one cause of immunosenescence and many aging related syndromes. This study provides evidence for the aging model of thyrotoxicosis mice, which can be used for exploring anti-aging drugs and strategies.

## Introduction

The enormous increase in the proportion of the elderly has brought a series of challenges to societies all over the world. The increased prevalence of aging related diseases, poor prognosis, and prolonged hospitalization have led to a decline in quality of life and huge medical expenditure, which have a considerable impact on individuals, families, and society. Diseases cause huge losses to the body. These include the disease itself, fluid loss, and stress caused by surgery and other treatments. The nutritional status has an important influence on the treatment and prognosis of diseases (1, 2). But for elderly inpatients, aging brings a decline in digestive function, which is reflected by a weakening of intestinal nutrient absorption, making them prone to malnutrition (3). This means that elderly inpatients have poorer baseline nutritional status, which is reflected by anemia, hypoproteinemia, hypocholesterolemia, and so on. All of these syndromes are closely related to the incidence rate, prognosis, and mortality during hospitalization (4–6).

Nutritional deficiencies weaken the immune system and increase the invasion, replication, and mutation of viruses, which was a susceptibility and severity factor for COVID-19 in the elderly (7–9). Beyond COVID-19, immunosenescence is also considered to be the main cause of increased susceptibility to infection, loss of control of persistent infections, poorer responses to vaccination, and lower capacity to mediate anti-cancer responses, which was associated with increased morbidity and increased mortality (10, 11). Leukopenia, especially agranulocytosis, is a complication of hyperthyroidism, which usually leads to severe illness, as well as severe secondary inflammation (12). Several aspects of neutrophil response are affected by normal aging, including traditional neutrophil functions, such as phagocytosis and oxidative outbreaks. Because neutrophils never divide and have little capacity for self-repair, they are susceptible to death when activated or damaged, so if there is not enough supplement there is a decline in quantity. Neutrophil immunosenescence reduced the ability to eliminate bacteria and fungi, inhibited the interaction with the adaptive immune system, and affected the adaptive immune system, leading to a decline in immunity (13). The decrease of the CD4+/CD8+ ratio is one of the markers of T cell “immunosenescence” (14). The most striking and extreme example of immunosenescence is AIDS, which is closely related to the prognosis of AIDS (15). Additionally, it is of great significance to evaluate the host immune function by combining the number and function of lymphocytes for the diagnosis, treatment, and prognosis of diseases (16). Ability to produce IFN-γ can be used as a marker of lymphocyte function, Th1 CD4 effector T cells can produce large amounts of IFN-γ to fight the infection of intracellular pathogens, thereby stimulating and maintaining effective cellular immune responses (17).

The decline of absorption and use of nutrients is a degenerative performance. The utilization of nutrients also needs functional proteins, for example, transporters and binding proteins. The loss of these functional proteins is also a feature of aging (18). In addition, in the aging process, the accumulation of harmful metabolites destroys the internal environment homeostasis and gradually leads to cell dysfunction and organ failure, such as lipotoxicity related metabolites (19). The internal environmental homeostasi**s** includes the relative stability of nutrients, metabolic waste, and other components. Other components include hormones, antibodies, and neurotransmitters, which are under precise regulation, minor changes will affect normal physiological functions, such as sex hormones on the menstrual cycle, and antibodies on immunity, which will decline with aging (20). Moreover, long-term or multiple high-intensity stress exposure will lead to stress-acceleration of aging, the reason might be due to the accumulation of metabolite toxicity such as stress hormones glucocorticoids (GCs) (21, 22).

Aging is a series of events that reflect cumulative damage across regulatory systems, including nutrition, metabolism, immunity, and endocrine. While simulating aging to explore new intervention strategies depends on the methods and tools used in the laboratory. But so far, no suitable animal model has been found to integrate the above factors. As well known, thyroid hormone (TH) plays a very important role in almost all body tissues by regulating metabolism, growth, and development (23), and thyroid hormone can dose-dependent increase the energy consumption of most tissues of the whole body, which is related to accelerated kinetics and accelerates the process of life (24). Studies have found that elevated thyroxine levels are associated with aging and shortened lifespan, and the longevity of vertebrate species is usually positively correlated with low metabolic rates and low TH levels (25, 26). We speculated that the thyrotoxicosis model might be an ideal accelerated aging model. Although many studies have used this model to study the mechanism of hyperthyroidism and the development of hyperthyroidism protective drugs, there is little discussion of models simulating aging, especially the relationship between hyperthyroidism with immunosenescence, for autoimmune factors of hyperthyroidism mask the real impact of thyroid hormones on the immune system. Current studies have focused on the thermogenic reaction of TH on brown and/or white adipose tissue, and there are few studies on the changes of metabolites in this process (27). Accelerating metabolism is bound to bring a series of chain reactions, which will lead to the changes in metabolites, and have a great impact on the internal environmental homeostasis. Exploring the changes of metabolites in this process will find more evidence for the model to simulate aging. In this study, on the basis of routine biochemical and hematological indicators, proteomics combined with metabonomics was used to analyze changes in serum proteome and endogenous metabolome as well as the fecal metabolome, mechanisms, and markers behind the relationship between thyrotoxicosis and aging.

## Materials and Methods

### Animals and Reagents

4-6 weeks old healthy female KM mice (SPF grade) with body weights of 16-18 g were purchased from Ji’nan Pengyue Laboratory Animal Breeding Co., Ltd (Shandong, China). The mice were housed in a clean room at a temperature of 23 ± 2°C and humidity of 50 ± 5% with a 12 h alternating light and dark cycle. They were permitted free access to food and water. All animal experiments were performed according to the National Institutes of Health Guidelines for the Care and Use of Laboratory Animals and were approved by the Animal Care and Use Committee of Shandong Province, China. The thyroxine tablets were purchased from Shandong Renhe Pharmaceutical Co., Ltd (China). PerCP-Cy™5.5 Hamster Anti-Mouse CD3e (Cat# 551163), FITC Rat Anti-Mouse CD4 (Cat# 553047), APC-H7 Rat anti-Mouse CD8a (Cat# 560182), PE Rat Anti-Mouse IFN-γ (Cat# 554412), Leukocyte Activation Cocktail, with BD GolgiPlug (Cat. No.550583) were purchased from BD Pharmingen™. All chemicals and solvents were analytical or HPLC grade water, methanol, acetonitrile, formic acid, pyridine, n-hexane, methoxylamine hydrochloride, N, O-Bis (trimethylsilyl) trifluoroacetamide (BSTFA) with 1% chlorosilane were purchased from CNW Technologies GmbH (Germany), L-2 chlorophenylalanine was from Shanghai Hengchuang Bio technology Co Ltd. (China).

### Thyrotoxicosis Model

**Figure 1:**
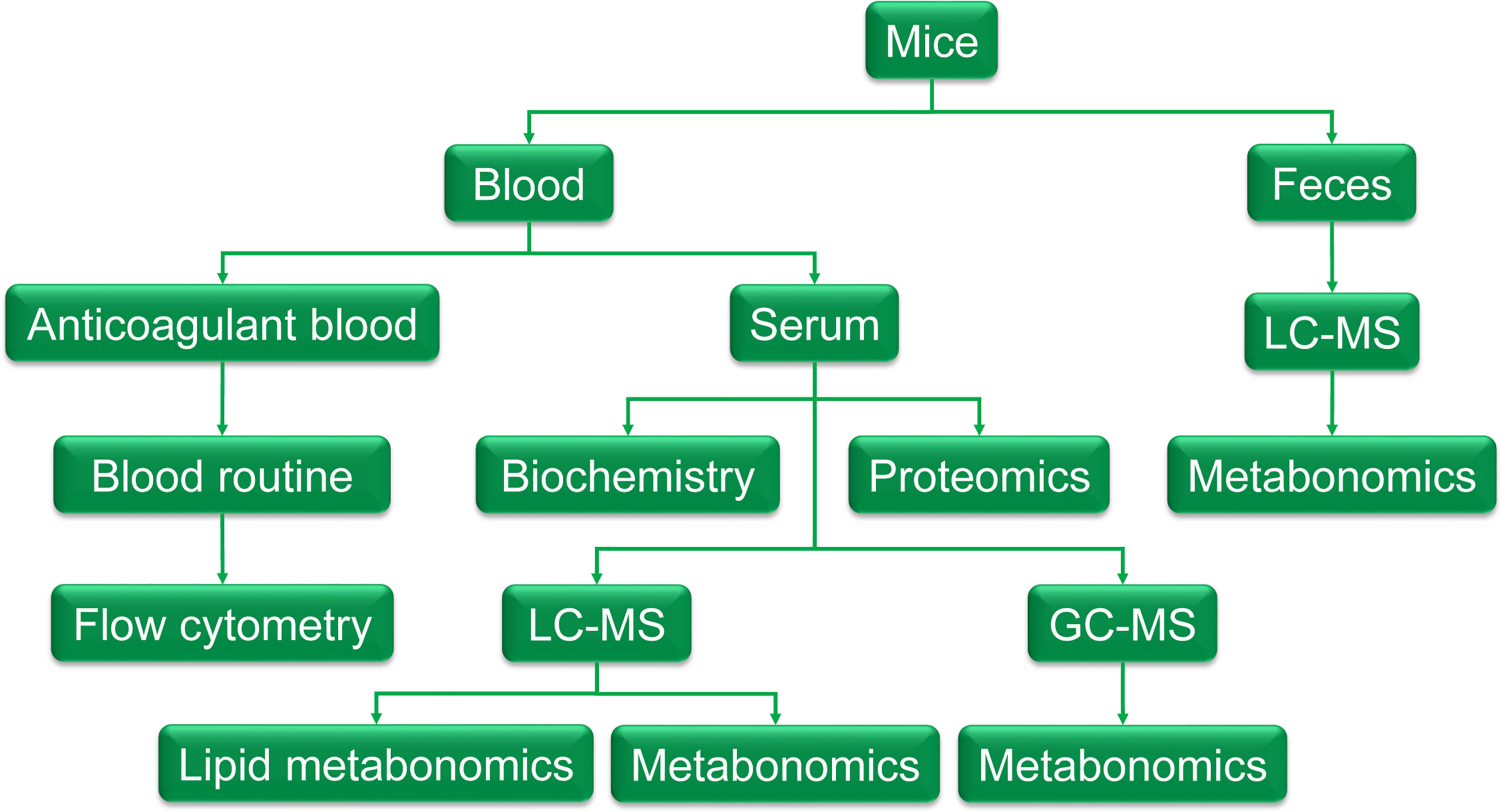
The graphic of the methodology. Feces were collected the day before the end of the experiment and was cryopreserved at - 80°C for metabolomic examination. At the end of the experiment, fasting the night before dissection, blood was taken from the main abdominal vein after anesthesia with 60mg·kg^-1^ pentobarbital sodium. About 300ul of blood was injected into EDTA-K2 vacuum tubes to get anticoagulant blood for a routine blood test and flow cytometry, the remaining blood was injected into a gel separation tube and the serum was separated after centrifugation of 1000g. The serum was taken out for biochemistry detection, proteomics, and metabolomics detection.

Mice were randomly divided into 2 groups: the control group (C) and the model group (T). Intragastric administration of thyroxine tablets suspension (320mg·kg^-1^), once a day, for 20 days. Feces were collected on day 20 and was cryopreserved at - 80°C for metabolomic examination. At the end of the experiment, fasting the night before dissection, blood was taken from the main abdominal vein after anesthesia with 60mg·kg^-1^ pentobarbital sodium. About 300ul blood was injected into EDTA-K2 vacuum tubes for routine blood tests and flow cytometry, the remaining blood was injected into a gel separation tube and the serum was separated after centrifugation of 1000g. The serum was taken out for biochemical detection, proteomics, and metabolomics detection. The methodology is shown in **Figure 1**.

### Routine Blood Tests

EDTAK2 anticoagulant whole blood was taken using routine blood tests. The routine blood tests were carried out by Sysmex XN-1000v [B1] automated hematology analyzer (SYSMEX Co., Ltd., Japan).

### Detection of CD4^+^, CD8^+^ T Lymphocytes

The positive rates of CD4^+^, CD8^+^ T lymphocyte subsets were analyzed by flow cytometry. Briefly, 3 mL of red blood cell lysis buffer was added to each sample to completely lyse red blood cells. Then the samples were washed and re-suspended with DMEM to 1 × 10^6^ cells/mL. Cells were transferred to 48-well plates with a 100 μL volume of each well, 2 μL of cell activation cocktail (BD biosciences) and 1 μL of BrefeldinA (BD biosciences) were added to each sample and incubated at 37°C for 6 h. After being washed and re-suspended with PBS, samples were incubated with specific fluorescent antibodies (BD biosciences) of PerCP-Cy™5.5 Hamster Anti-Mouse CD3e (0.5 μl/sample), FITC Rat Anti-Mouse CD4 (0.2 μl/sample), and APC-H7 Rat anti-Mouse CD8a (0.5 μl/sample) for 30 min at room temperature in the dark according to the manufacturer’s guidelines. All samples were stained in triplicate. The samples were analyzed by the CytoFLEX flow cytometer (Beckman Coulter Life Sciences), and the data were analyzed by the CytExpert software (Beckman Coulter Life Sciences).

### Detection of CD4^+^IFN-γ^+^ T Lymphocytes

To further determine the levels of CD4^+^IFN-γ^+^ T lymphocyte subsets, cells treated in the previous step were fixed with 500 μL of 4% FA for 20 min in the dark, ruptured with 1 mL of Permeabilization Wash Buffer (BD biosciences). Then the cells were stained with PE Rat Anti-Mouse IFN-γ (1.25 μg/sample) for 30 min. All samples were stained in triplicate. The samples were analyzed by the CytoFLEX flow cytometer (Beckman Coulter Life Sciences), and the data were analyzed by the CytExpert software (Beckman Coulter Life Sciences).

### Serum Biochemical Analysis

Serum biochemical analyses were detected by BS-800 automatic biochemistry analyzer (Shenzhen Mindray Bio-Medical Electronics CO., Ltd., China).

### Measurement of Serum Proteome Using DIA-MS

40 ul serum for each sample was used for proteomics analysis. Briefly, the total protein was extracted and removed albumin/IgG. Protein concentration was determined by the BCA method. 10 μg protein of each sample was separated by 12% SDS-PAGE. The digested peptides were desalted by the C18-Reverse-Phase SPE Column. RP separation was performed on an 1100 HPLC System (Agilent) using an Agilent Zorbax Extend RP column (5 μm, 150 mm × 2.1 mm). The separated peptides were lyophilized for mass spectrometry. All analyses were performed by a Q-Exactive HF mass spectrometer (Thermo, USA) equipped with a Nanospray Flex source (Thermo, USA). The machine signal is transformed into peptide and protein sequence information by matching the mass spectrum output with the theoretical spectrum generated by fasta library, and then the spectrum DDA (Data-dependent Acquisition) library is established by combining the sequence information, peptide retention time, and fragment ion information, to facilitate the subsequent Data Independent Acquisition (DIA) analysis. The original LC-MS/MS files are imported into Spectronaut Pulsar software to search and build the database. The original data of DIA is processed by Spectronaut Pulsar software.

### Measurement of Serum Metabolome Using LC-MS

120 μL serum of each sample was added to a 1.5 mL Eppendorf tube with internal standard. The ice-cold mixture of methanol and acetonitrile (2:1 v/v) was then added to precipitate protein. The tube was centrifuged and the final supernatant was filtered through 0.22 μm microfilters and transferred to LC vials. QC samples were prepared by mixing aliquots of all 18 samples to be a pooled sample. The vials were stored at 80°C until LC-MS analysis. An ACQUITY UHPLC system (Waters Corporation, Milford, USA) coupled with an AB SCIEX Triple TOF 5600 System (AB SCIEX, Framingham, MA) was used to analyze the metabolic profiles in both ESI positive and ESI negative ion modes. An ACQUITY UPLC BEH C18 column (100 mm × 2.1mm, 1.7 μm) was employed in both positive and negative modes. The QCs were injected at regular intervals (every 6 samples) throughout the analytical run to provide a set of data from which repeatability can be assessed. Metabolites were identified by progenesis QI (Waters Corporation, Milford, USA) Data Processing Software, based on public databases such as Human Metabolome Database (http://www.hmdb.ca/)and LIPID MAPS Structure Database (http://www.lipidmaps.org/).

### Measurement of Serum Metabolome Using GC-MS

80 μL of the sample was added to a 1.5 mL Eppendorf tube with internal standard. Subsequently, the ice-cold mixture of methanol and acetonitrile (2/1, v/v) was added and centrifuged. The QC sample was prepared by mixing aliquots of all samples into a pooled sample. An aliquot of the 150 μL supernatant was transferred to a glass sampling vial for vacuum drying at room temperature. And 80 μL of 15 mg/mL methoxylamine hydrochloride in pyridine was subsequently added. The resultant mixture was vortexed vigorously for 2 min and incubated at 37°C for 90 min. 80 μL of BSTFA (with 1% TMCS) and 20 μL n hexane were added to the mixture, which was vortexed vigorously for 2 min and then derivatized at 70°C for 60 min. The samples were placed at ambient temperature for 30 min before GC MS analysis. The derivatized samples were analyzed on an Agilent 7890B gas chromatography system coupled to an Agilent 5977A MSD system (Agilent Technologies Inc., CA, USA). ADB-5MS fused-silica capillary column (30m×0.25mm×0.25μm, Agilent J&W Scientific, Folsom, CA, USA) was utilized to separate the derivatives. Helium (>99.999%) was used as the carrier gas at a constant flow rate of 1 mL/min through the column. The QCs were injected at regular intervals (every 6 samples) throughout the analytical run to provide a set of data from which repeatability could be assessed. Metabolites were identified by progenesis QI (Waters Corporation, Milford, USA) Data Processing Software, based on public databases such as Human Metabolome Database.

### Measurement of Fecal Metabolome Using LC-MS

60mg fecal of each sample was added to a 1.5 mL Eppendorf tube with internal standard, and then the ice-cold mixture of methanol and water (4:1 v/v) was added to each sample. Samples were stored at -20°C for 5 min and then ground at 60 HZ for 2 min, ultrasonicated in an ice water bath for 10 min, and stored at -20°C for 30 min. The extract was centrifuged at 13000 rpm, 4°C for 15 min. 300 μL of supernatant in a brown and glass vial was dried in a freeze concentration centrifugal dryer. 400 μL mixture of methanol and water (1:4, v/v) were added to each sample, samples vortexed for 30 s, then placed at -20°C for 2 h. Samples were centrifuged at 13000 rpm, 4°C for 10 min. The supernatants (150 μL) from each tube were collected using crystal syringes, filtered through 0.22 μm microfilters, and transferred to LC vials. The vials were stored at -80°C until LC-MS analysis. QC samples were prepared by mixing aliquots of all 18 samples to be a pooled sample. A Dionex Ultimate 3000 RS UHPLC system fitted with Q Exactive quadrupole Orbitrap mass spectrometer equipped with heated electrospray ionization (ESI) source (Thermo Fisher Scientific, Waltham, MA, USA) was used to analyze the metabolic profiling in both ESI positive and ESI negative ion modes. An ACQUITY UPLC HSS T3 column (100 mm × 2.1mm, 1.8 μm) was employed in both positive and negative modes. The QCs were injected at regular intervals (every 6 samples) throughout the analytical run to provide a set of data from which repeatability can be assessed. Metabolites were identified by progenesis QI (Waters Corporation, Milford, USA) Data Processing Software, based on public databases such as Human Metabolome Database (http://www.hmdb.ca/).

### Data and Statistical Analysis

All experimental data obtained from rats were expressed as mean ± SD. A one-way repeated measure analysis of variance (ANOVA) and a log-rank test were used to determine the significance of the differences in differential blood count, biochemical index, subsets, and function of lymphocytes, respectively.

For DIA proteomics data, statistical analyses and plots were performed using R Studio software version 1.1.463 (RStudio, Inc.). Using the database to retrieve the original data and keep any set of samples with proteins whose expression value accounts for ≥50%. The protein with a missing value <50% is filled in with the mean value of the same group of samples, and the credible protein is obtained through log10 conversion. Principal component analysis (PCA) was performed using the expression of a trusted protein. Based on the trusted protein, two standards are selected to calculate the difference between samples. Among them, Fold change is used to evaluate the fold change in the expression level of a certain protein between samples; The p-value calculated by the T-test test shows the significance of the difference between samples. Difference screening conditions: log10 Foldchange (FC) =1 and p-value < 0.05. log10 FC>1 was considered to be upregulated proteins, while log10 FC<1 was considered to be downregulated ones. R package was used for the bioinformatics analysis of differentially expressed proteins (DEPs), and these analyses included Metascape online analysis (https://metascape.org/), and String online database (https://string-db.org/) and KEGG pathway classification.

For LC-MS and GC-MS, differentially expressed metabolites (DEMs) were selected on the basis of the combination of a statistically significant threshold of variable influence on projection (VIP) values obtained from the (orthogonal) partial least-squares-discriminant analysis (OPLS-DA) model and p values from a two tailed Student’s t test on the normalized peak areas from different groups, where metabolites with VIP values larger than 1.0 and p values less than 0.05 were considered as differential metabolites.

Correlation analysis used the Pearson correlation coefficient to measure the linear correlation between two quantitative variables. Correlation significance is presented by the p value.

## Results

### Changes in Differential Blood Count and Biochemical Index

Blood routine test results showed that the total number of white blood cells (WBC), neutrophils (NEUT), lymphocytes (LYMPH), red blood cells (RBC), hemoglobin (HGB), Hematocrit (HCT), and platelets (PLT) were all decreased, which suggested that pancytopenia was induced by excessive thyroxine in mice (**Table 1**).

**Table 1:** Changes in differential blood count and biochemical index in mice with thyrotoxicosis.

		C ( n= 10)	T (n = 10)
WBC	10^9^/L	3.45 ± 0.99	2.07 ± 0.30^##^
NEUT	10^9^/L	0.50 ± 0.25	0.32 ± 0.13^#^
LYMPH	10^9^/L	2.65 ± 0.87	1.45 ± 0.24^##^
EO	10^9^/L	0.16 ± 0.10	0.08 ± 0.03^#^
RBC	10^12^/L	9.36 ± 0.42	8.76 ± 0.35^##^
HGB	g/L	137.80 ± 4.52	127.30 ± 6.80^##^
HCT	%	41.45 ± 1.64	39.73 ± 1.95^#^
RDW-SD		21.98 ± 2.20	26.30 ± 2.11^##^
RDW-CV		15.60 ± 2.03	17.78 ± 0.92^##^
PLT	10^9^/L	843.70 ± 85.56	692.50 ± 53.84^##^
ALT	U/L	30.00 ± 10.92	70.10 ± 20.92^##^
AST	U/L	51.85 ± 8.89	119.10 ± 27.87^##^
TP	g/L	42.44 ± 2.13	35.60 ± 2.28^##^
ALB	g/L	21.67 ± 1.91	17.25 ± 1.44^##^
GLB	g/L	20.78 ± 1.86	18.06 ± 1.29^##^
TG	mmol/L	1.93 ± 0.44	2.49 ± 0.73^#^
TC	mmol/L	2.37 ± 0.51	1.62 ± 0.32^##^
LDC-C	mmol/L	0.21 ± 0.07	0.13 ± 0.03^##^
HDL-C	mmol/L	1.94 ± 0.42	1.3 ± 0.26^##^

C, normal control mice; T, thyrotoxicosis mice. Compared with normal control mice ^#^p < 0.05, ^##^p < 0.01.

Biochemical analysis showed that the levels of Alanine aminotransferase (ALT) and Aspartate aminotransferase (AST) increased significantly after 20 days of continuous intake of overdose thyroxine, suggesting that these mice had liver damage. The levels of Total protein (TP), Albumin **(**ALB), and Globulin (Glb) were significantly decreased, which indicated that excessive thyroxine induced hypoproteinemia or hypoalbuminemia. The levels of total cholesterol (TC), Low-density lipoprotein cholesterol (LDC-C), and High-density lipoprotein cholesterol (HDL-C) were significantly decreased, while triglyceride (TG) was significantly increased, indicating that thyrotoxicosis mice had hypocholesterolemia, hypolipoproteinemia, and hyperlipidemia.

### Changes in Subsets and Function of Lymphocytes

**Figure 2:**
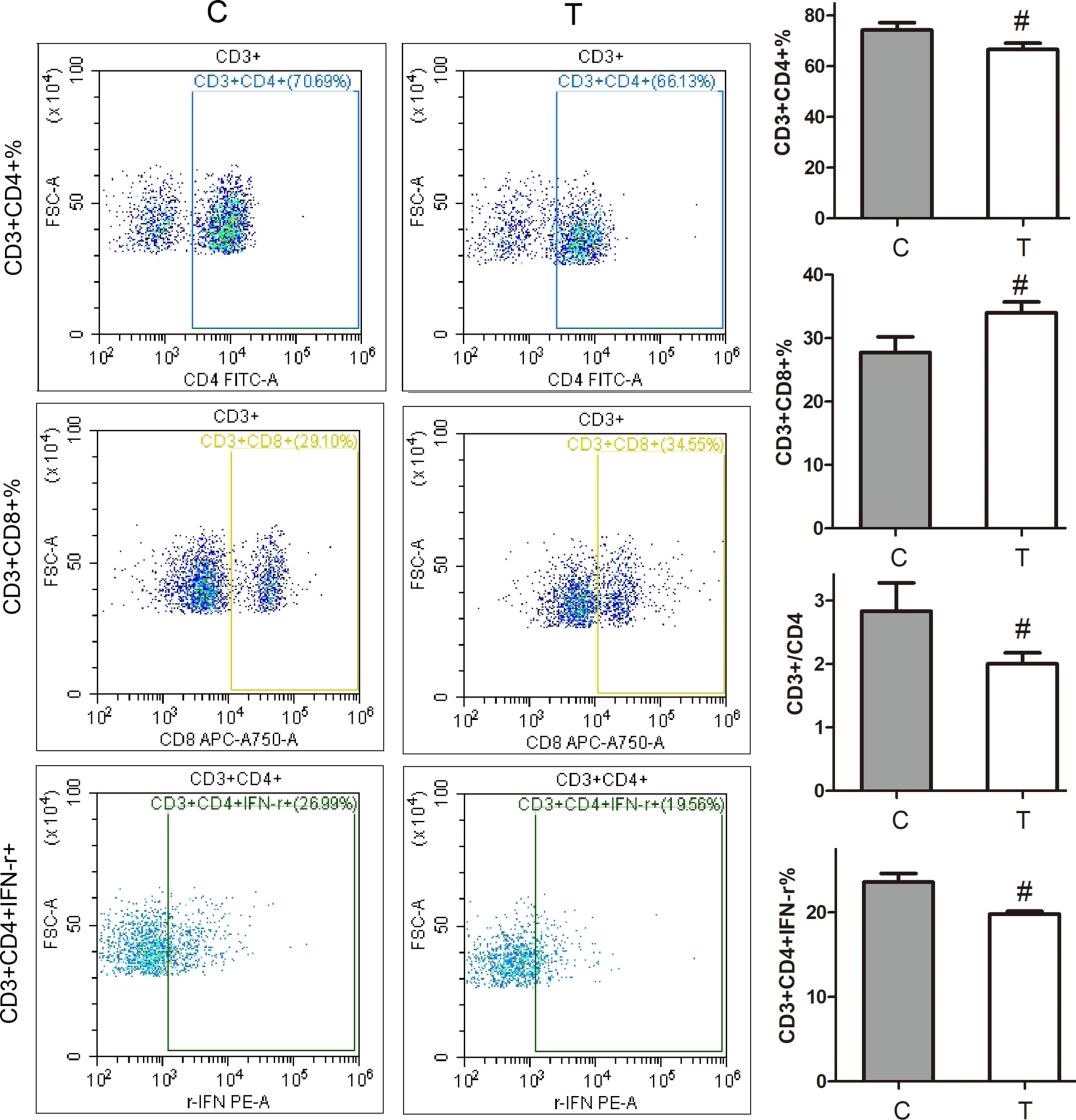
Changes in subsets and function of lymphocytes. Compared with the normal control group, the percentage of CD3+CD4+ in thyrotoxicosis mice decreased significantly, while the percentage of CD3+CD8+ increased significantly, so the percentage of CD4+/CD8+ decreased significantly. The percentage of CD3^+^CD4^+^ IFN-r^+^ was significantly decreased in thyrotoxicosis mice. C, normal control group; T, thyrotoxicosis group. Compared with normal control mice #p < 0.05.

As shown in **Figure 2**, compared with the normal control group, the percentage of CD3+CD4+ in thyrotoxicosis mice decreased significantly, while the percentage of CD3+CD8+ increased significantly, so the percentage of CD4+/CD8+ decreased significantly. The percentage of CD3^+^CD4^+^ IFN-**γ**^+^ was significantly decreased in thyrotoxicosis mice.

### Comparative Serum Proteomic Analysis Between Thyrotoxicosis Mice and Normal Mice

**Figure 3:**
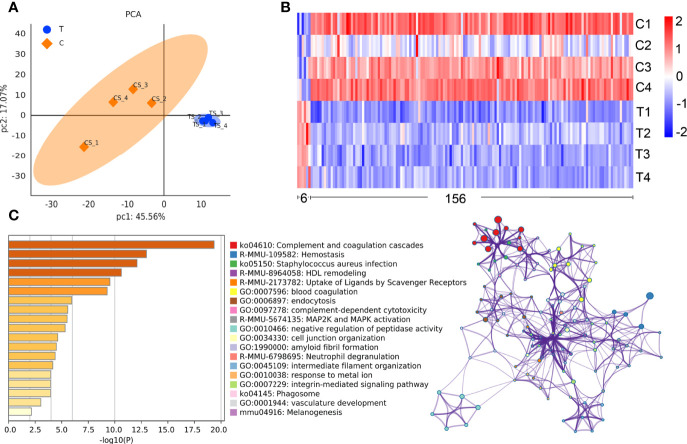
Comparative proteomic analysis. **(A)** PCA analysis showed that there was a significant difference between the two groups. **(B)**The heat map of the differential proteins: The difference screening condition was log10 foldchange = 1 times and p-value < 0.05. The DEPs with foldchange<1 times were selected as downregulated proteins, that of >1 times were selected as upregulated proteins, including 6 upregulated ones and 156 downregulated ones. **(C)** The top enrichment 20 terms according to -log10 p value made by Metascape online analysis.

Using the expression of believable protein for principal component analysis (PCA analysis), there was a significant difference between the two groups (**Figure 3A**). A total of 779 proteins (DDA library) were identified in this study, among which 351 proteins were quantitated. Compared with normal control mice, the proteomic analysis highlighted 162 differentially expressed proteins (DEPs) in thyrotoxicosis mice, among them, 6 were up-regulated and 156 were downregulated (**Figure 3B**). 156 downregulated proteins were analyzed by Metascape online analysis. The top 20 enrichment terms were Complement and coagulation cascades, Hemostasis, Staphylococcus aureus infection, HDL remodeling, Uptake of ligands by scavenger receptors, Blood coagulation, endocytosis, complement-dependent cytotoxicity, MAP2K and MAPK activation, Negative regulation of peptidase activity, Cell junction organization, Amyloid fibril formation, Neutrophil degranulation, Intermediate filament organization, Response the metal ion, Integrin-mediated signaling pathway, Phagosome, Vasculature development, and Melanogenesis (**Figure 3C**).

**Figure 4:**
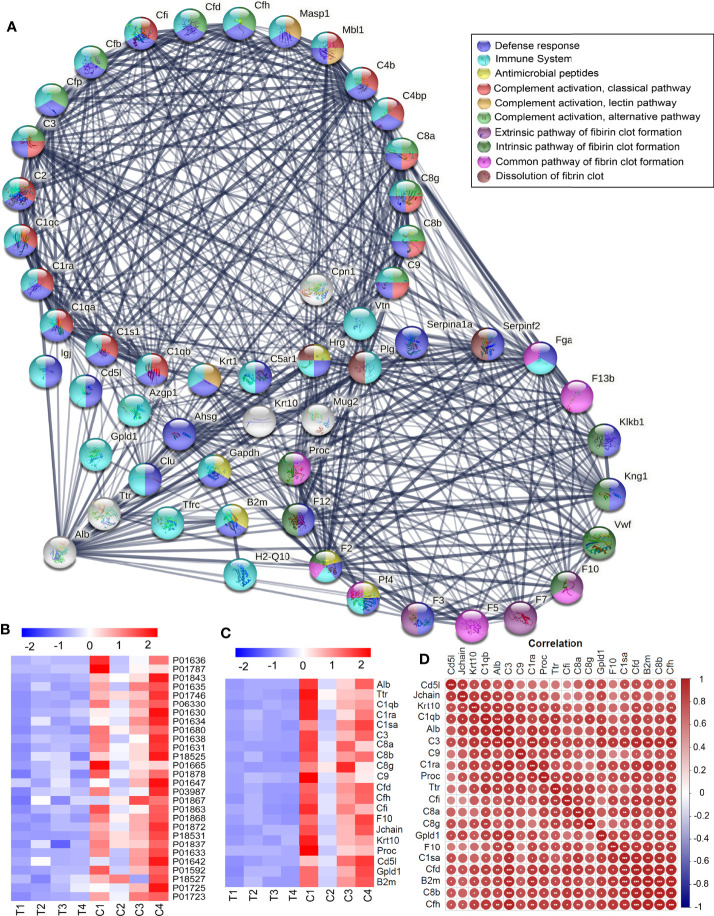
Downregulated DEPs in Complement and coagulation cascades. **(A)** The downregulated DEPs in the Complement cascade covered all three complement activation pathways: alternative pathway, classical pathway, and lectin pathway. The blood coagulation pathways were downregulated, including common pathway, intrinsic pathway, and extrinsic pathway, and anticoagulation and fibrinolysis factors were also downregulated, such as Plg, Serpinf2, Serpina10, Proc, and Hrg. Of these down regulated proteins, 32 were related to the immune system, including complement components and 4 antimicrobial peptides Hrg, F2, Pf4, and B2m, and most of them were involved in defense response. **(B)** A large number of immunoglobulins were downregulated in thyrotoxicosis mice. **(C)** The correlation analysis was carried out for Alb and Trf and some DEPs in complement and coagulation cascades, it showed that Alb was positively correlated with those DEPs in complement and coagulation cascades. Red is positive correlation, correlation significance: *p<0.05; **p<0.01; ***p<0.001.

The STRING online analysis (https://string-db.org/) displayed the detail of complement and coagulation cascades (**Figure 4A**), as well as Alb and Transthyretin (Ttr). DEPs in the Complement cascade covered all three complement activation pathways: alternative pathway, classical pathway, and lectin pathway. It was also found that the blood coagulation pathways were downregulated, including common pathway, intrinsic pathway, and extrinsic pathway, and anticoagulation and fibrinolysis factors were also downregulated, such as Plg, Serpinf2, Serpina10, Proc, and Hrg. Of these downregulated proteins, 32 were related to the immune system, including complement components and 4 antimicrobial peptides Hrg, F2, Pf4, and B2m, which suggested that humoral immune response was declined. In addition, there were a large number of immunoglobulins downregulated, indicating that the adaptive immune system also declined (**Figure 4B**). The correlation analysis was carried out for Alb and Trf and some DEPs in complement and coagulation cascades, it showed that Alb was positively correlated with those DEPs in complement and coagulation cascades (**Figures 4C, D**).

**Figure 5:**
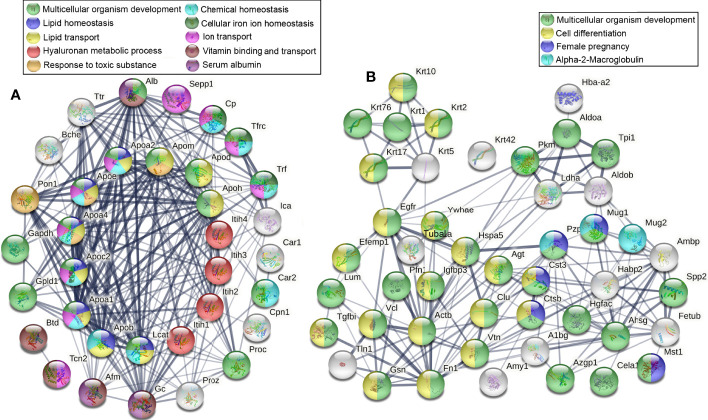
Downregulated DEPs related to homeostasis and multicellular organism development. **(A)** The DEPs that are related to lipid, ions, and chemical homeostasis accounted for lipid transport, ion transport, vitamin transport, and PH maintenance, their downregulation seriously affected the system multicellular organism development of thyrotoxicosis mice. **(B)** The DEPs directly related to multicellular organism development, many of them were involved in the cell differentiation process and female pregnancy.

We continue to analyze the remaining downregulated DEPs by STRING online analysis, and found that many DEPs were related to homeostasis, they accounted for lipid transport, ion transport, and vitamin transport. Their downregulation indicated the destruction of the homeostasis of lipid, ions, and chemicals, which would seriously affect the system multicellular organism development of thyrotoxicosis mice (**Figure 5A**). More than that, a lot of downregulated DEPs were directly related to multicellular organism development, many of them were involved in the cell differentiation process, even female pregnancy (**Figure 5B**). Their downregulations indicated thyroxine poisoning seriously affected system development and reproduction.

### Results of Serum LC-MS Metabolomics Analysis and the Correlation With Proteomics

**Figure 6:**
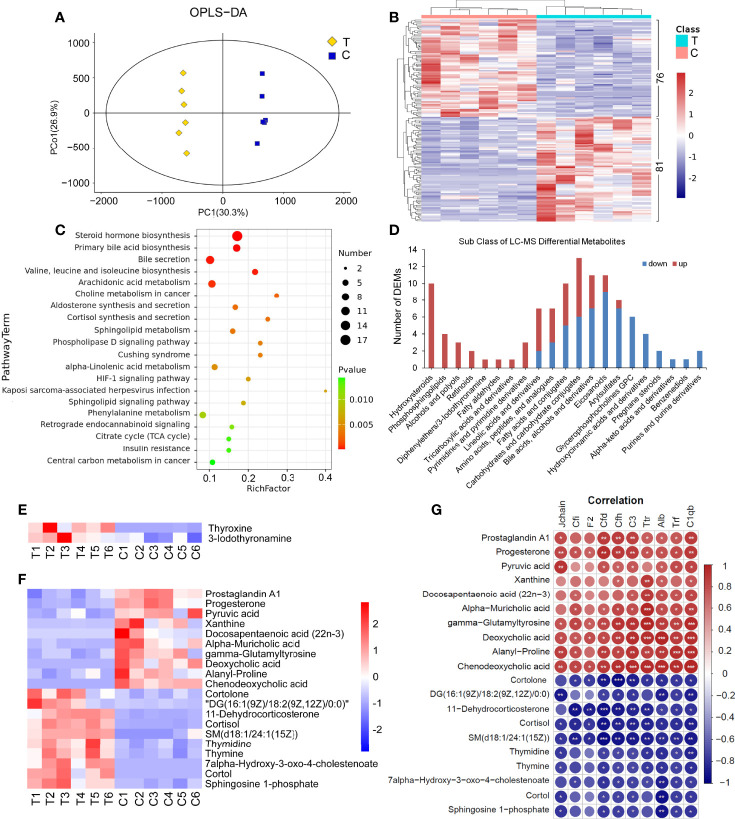
Serum LC-MS metabolomics analysis and the correlation with proteomics. **(A)** There were significant differences in OPLS-DA score between the thyrotoxicosis group (T) and the normal control group **(C)**. OPLS-DA, orthogonal partial least squares discrimination analysis. **(B)** Clustering heat map of 157 DEMs, including 81 upregulated ones and 76 downregulated ones (HMDB database). **(C)** The KEGG pathway analysis result of all DEMs. **(D)** The classification of LC-MS DEMs. **(E)** The expression of Thyroxine (T4) and Iodothyronamine (T3) were significantly upregulated in thyrotoxicosis mice. **(F, G)** 20 DEMs that were significantly correlative with Alb and C1qb. Red is positive correlation and blue is negative correlation, correlation significance: *p<0.05; **p<0.01; ***p<0.001.

There were significant differences in the OPLS-DA score between the thyrotoxicosis group and the normal group (**Figure 6A**). Based on Human Metabolome Database (HMDB), LC-MS-based metabolomics identified and quantified 821 metabolites in serum, of which 157 were differentially expressed metabolites (DEMs), including 81 upregulated and 76 downregulated (**Figure 6B**). KEGG pathway analysis showed that these DEMs were enriched in the pathway of Steroid hormone biosynthesis, Primary bile acid biosynthesis, Bile secretion, Valine, leucine, and isoleucine biosynthesis, Arachidonic acid metabolism, Sphingolipid signaling pathway, and so on (**Figure 6C**). The classification of DEMs was displayed in **Figure 6D**. “Hydroxyl steroid” corresponded to “Steroid hormone biosynthesis”, “Phosphosphingolipids” corresponded to “Sphingolipid signaling pathway”, and the DEMs in these two classes were all upregulated. “Bile acids, alcohols and derivatives” corresponding to “Primary bile acid biosynthesis” and “Bile secretion”, there are 11 DEMs in this class, of them 7 were downregulated. “Eicosanoids’ corresponded to “Arachidonic acid metabolism”, in this class 9 of 11 were downregulated. The DEMs in “Fatty acids and conjugates” and “Lineolic acids and derivatives” classes had ups and down. 7 of 8 DEMs in “Arylsulfates” class were downregulated. The DEMs of “Glycerophosphocholines (GPC)”, “Hydroxycinnamic acids and derivatives”, “Pregnane steroids”, and “Purines and purine derivatives” were all downregulated.

It is noteworthy that significant increases in Thyroxine (T4, FC=12.72, *p*=0.0016) and 3-Iodothyronamine (T3, FC=2.41, *p*=0.0394) were also detected by LC-MS (**Figure 6E**), which proved the occurrence of thyrotoxicosis.

We highlighted some DEPs in Complement and coagulation cascades, such as Cfi, Cfd, Cfh, C3, C1qb, F2, Jchain, as well as Alb, Trf, and Ttr. The correlation analysis between the above DEPs and all DEMs in serum was carried out. On the condition that the correlations with Alb and C1qb were significant at the same time, 20 DEMs were screened out in LC-MS DEMs (**Figure 6F**). As shown **in** **Figure 6G**, among them, the downregulated DEMs were positively correlated with these downregulated DEPs, including Prostaglandin A1, Progesterone, Pyruvic acid, Xanthine, Docosapentaenoic acid (22n-3) (DPA), Alpha-Muricholic acid, gamma-Glutamyltyrosine, Deoxycholic acid, Alanyl-Proline, and Chenodeoxycholic acid; the upregulated DEMs were negatively correlated with these downregulated DEPs, including Cortolone, DG(18:2(9Z,12Z)/18:2(9Z,12Z)/0:0), 11-Dehydrocorticosterone, Cortisol, SM(d18:1/24:1(15Z)), Thymidine, Thymine, 7alpha-Hydroxy-3-oxo-4-cholestenoate, Cortol, and Sphingosine 1-phosphate.

### Results of Serum Lipid Metabolomics Analysis and the Correlation With Proteomics

**Figure 7:**
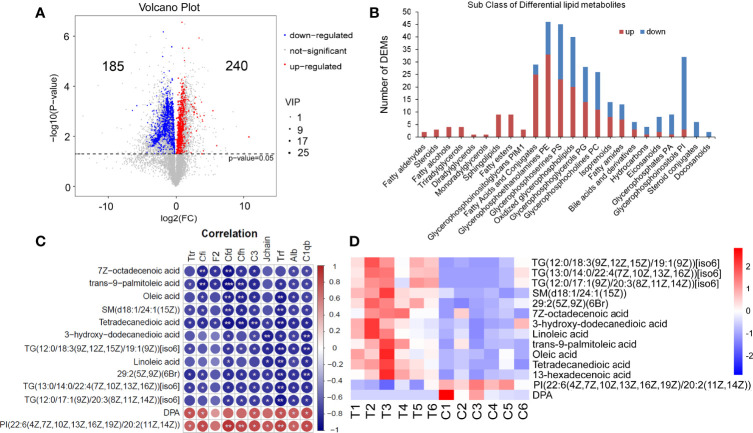
Serum lipid metabolomics analysis and the correlation with proteomics. **(A)** The Volcano plot of 325 lipid DEMs (Lipidmaps Database), including 240 upregulated ones and 185 downregulated ones. **(B)** The classification of LC-MS lipid DEMs (Lipidmaps Database). **(C, D)** 13 DEMs that were significantly correlative with Alb and C1qb. T, thyrotoxicosis mice; C, normal control mice. Red is positive correlation and blue is negative correlation, correlation significance: *p<0.05; **p<0.01; ***p<0.001.

Based on Lipidmaps (v2.3) Database, LC-MS-based metabolomics identified and quantified 3333 lipid metabolites in serum, of which 425 were differentially expressed metabolites (DEMs), including 240 upregulated and 185 downregulated (**Figure 7A**). The classification of these DEMs is displayed in **Figure 7B**. Among them, All Fatty aldehydes, Ceramides, Steroids, Fatty alcohols, Monoradylglycerols, Diradylglycerols, Triradylglycerols, Fatty esters, Glycerophosphoinositolglycans PIM1, 25 of 29 Fatty Acids and Conjugates were upregulated. The DEMs in Glycerophosphoethanolamines (PE), Glycerophosphoserines (PS), Oxidized glycerophospholipids, Glycerophosphoglycerols (PG), Glycerophosphocholines (PC), Isoprenoids, Fatty amides, and Bile acids and derivatives had ups and downs. Hydrocarbons, Eicosanoids, Glycerophosphates (PA), and Glycerophosphoinositols (PI) are mainly downregulated. The DEMs in Steroid conjugates and Docosanoids classes were all downregulated.

The correlation analysis between lipid DEMs and above DEPs was performed. On the condition that the correlations with Alb and C1qb were significant at the same time, 13 lipid DEMs were screened out (**Figure 7C**). Among them, the downregulated DEMs were positively correlated with downregulated DEPs, including DPA and PI. The upregulated DEMs were negatively correlated with these downregulated DEPs, including TG(12:0/18:3(9Z,12Z,15Z)/19:1(9Z))[iso6], TG(13:0/14:0/22:4(7Z,10Z,13Z,16Z))[iso6], TG(12:0/17:1(9Z)/20:3(8Z,11Z,14Z))[iso6], SM(d18:1/24:1(15Z)), 29:2(5Z,9Z)(6Br), 7Z-octadecenoic acid, 3-hydroxy-dodecanedioic acid, Linoleic acid, trans-9-palmitoleic acid, Oleic acid and Tetradecanedioic acid. The heat map of these 13 DEMs is shown in **Figure 7D**.

### Results of Serum GC-MS Metabolomics Analysis and the Correlation With Proteomics

**Figure 8:**
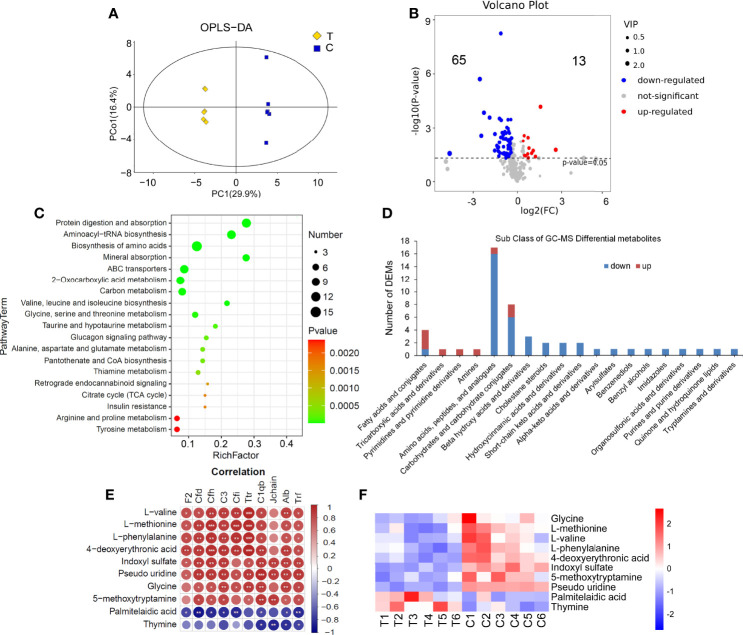
Serum GC-MS metabolomics analysis. **(A)** There were significant differences in OPLS-DA score between the thyrotoxicosis group (T) and the normal control group (C). **(B)** The Volcano plot of 80 lipid DEMs (HMDB database), including 13 upregulated ones and 65 downregulated ones. **(C)** The KEGG pathway analysis result of all DEMs. **(D)** The classification of GC-MS DEMs. **(E, F)** 13 DEMs that were significantly correlative with Alb and C1q. T, thyrotoxicosis mice; C, normal control mice. Red is positive correlation and blue is negative correlation, correlation significance: *p<0.05; **p<0.01; ***p<0.001.

There were significant differences in the OPLS-DA score between the thyrotoxicosis group and the normal group (**Figure 8A**). GC-MS-based metabolomics identified and quantified 885 metabolites in serum, of which 80 were differentially expressed metabolites (DEMs) (**Figure 8B**), including 13 upregulated and 67 downregulated. KEGG pathway analysis showed that these DEMs were enriched in the pathway of Protein digestion and absorption, Aminoacyl-tRNA biosynthesis, Biosynthesis of amino acids, Mineral absorption, ABC transporters, and so on (**Figure 8C**). These pathways are all amino acids related. The classification of these DEMs were displayed in **Figure 8D**. Most amino acids, peptides, and analogues, Carbohydrates and carbohydrate conjugates, Beta hydroxy acids and derivatives, and Cholestane steroids were mainly downregulated. In total, 3 of the 4 fatty acids and conjugates, Tricarboxylic acids and derivatives, Pyrimidines and pyrimidine derivatives, and Amines were upregulated.

We also analyzed the correlation between all DEMs that abstained from GC-MS and the above DEPs. On the condition that the correlations with Alb and C1qb were significant at the same time, 10 lipid DEMs were screened out (**Figure 8E**). Among them, the downregulated DEMs were positively correlated with those downregulated DEPs, including Glycine, L-methionine, L-valine, L-phenylalanine, 4-deoxyerythronic acid, Indoxyl sulfate, 5-methoxytryptamine, and Pseudo uridine, the upregulated DEMs were negatively correlated with these downregulated DEPs, including Palmitelaidic acid and Thymine. The heap map of these 10 DEMs is shown in **Figure 8F**.

### Results of Feces LC-MS Metabolomics Analysis

**Figure 9:**
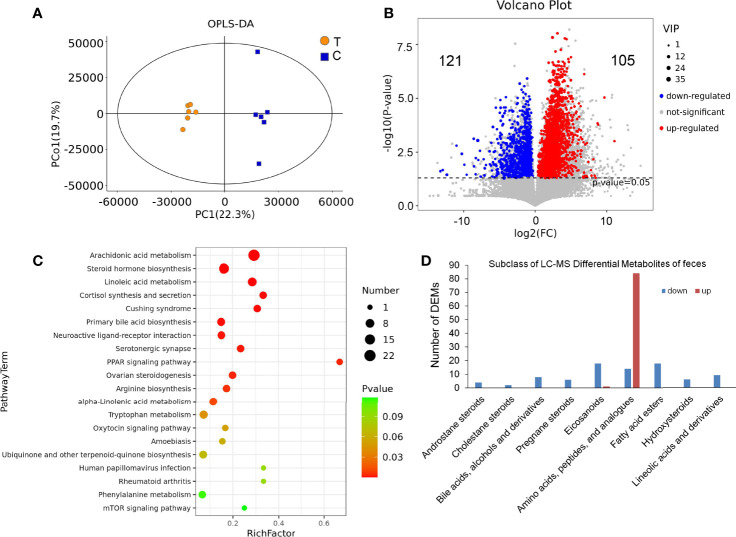
Feces LC-MS metabolomics analysis. **(A)** There were significant differences in OPLS-DA score between the thyrotoxicosis group (T) and the normal group (C). **(B)** The Volcano plot of 226 DEMs (HMDB database), including 105 upregulated ones and 121 downregulated ones. **(C)** The KEGG pathway analysis result of all DEMs. **(D)** The classification of LC-MS DEMs.

There were significant differences in the OPLS-DA score between the thyrotoxicosis group and the normal group (**Figure 9A**). Based on Human Metabolome Database (HMDB), LC-MS-based metabolomics identified and quantified 1820 metabolites in serum, of which 226 were differentially expressed metabolites (DEMs) (VIP > 1 and *p*-value value < 0.05), including 105 upregulated and 121 downregulated (**Figure 9B**). KEGG pathway analysis showed that these DEMs were enriched in the pathway of Arachidonic acid metabolism, Steroid hormone biosynthesis, Linoleic acid metabolism, Cortisol synthesis and secretion, Cushing syndrome, Primary bile acid biosynthesis, Neuroactive ligand-receptor interaction, and so on (**Figure 9C**), while the classification of these DEMs showed that the subclass of Amino acids, peptides, and analogues was the largest and most of them was upregulated. It also showed that the Androstane steroids, Cholestane steroids, Bile acids, Alcohols and derivatives, Pregnane steroids, Eicosanoids, Fatty acid esters, Hydroxysteroids, Lineolic acids, and derivatives were significantly downregulated in the feces of thyrotoxicosis mice (**Figure 9D**). Because most DEMs were dipeptides, they might be not able to be analyzed by KEGG analysis, so they are not shown in **Figure 9C**. In principle, these dipeptides should be listed in the pathway of Protein digestion and absorption, and their upregulation in feces suggested that these amino acids and dipeptides were lost with fecal excretion, which was the reason for hypoproteinemia.

### Consistency and Accuracy of Serum MS Results

DEMs in serum detected simultaneously by LC-MS and GC-MS included Pyruvic acid, Indoxyl sulfate, Xanthine, Oleic acid, Thymine, and Isocitric acid. The results of these DEMs in two methods are consistent, Pyruvic acid, Indoxyl sulfate, and Xanthine are all downregulated, while Oleic acid, Thymine, and Isocitric acid are all upregulated. The upregulated TGs detected by LC-MS, the downregulated Cholesterol detected by GC-MS and the downregulated Alb detected by LC-MS/MS were consistent with their results in the biochemical test (**Table 2**). Apoa1, Apoa2, and Apoa4 in DEPs represented HDL, and their downregulations were agreed with the result of HDL-C in the biochemical test. In total, 25 immunoglobulins or fragments were found downregulated in DEPs, Jchain, Igkc, and Ighg were selected as their representatives, and the results were consistent with that of Glb in the biochemical test. All the comparisons proved the accuracy of MS detection results.

**Table 2:** The Consistency of DEMs and DEP with different detection methods.

Metabolites	Compound ID	Fold Change (FC)		
		LC-MS	GC-MS	Biochemical Test
Pyruvic acid	HMDB0000243	0.36	0.63	
Indoxyl sulfate	HMDB0000682	0.02	0.17	
Xanthine	HMDB0000292	0.02	0.04	
Oleic acid	LMFA01030002/HMDB0000207	2.46	1.38	
Thymine	HMDB0000262	2.11	1.31	
Isocitric acid	HMDB0000193	2.08	1.62	
Cholesterol	HMDB0000067		0.70	0.68
TG(18:3/20:0/22:6)	LMGL03016429		2.22	1.29
TG(12:0/18:3/19:1)	LMGL03013521		6.20	
TG(13:0/14:0/22:4)	LMGL03013699		6.74	
TG(12:0/17:1/20:3)	LMGL03013408		7.78	
HDL-C Apoa1 Apoa2 Apoa4	Q00623 P09813 P06728	0.22 0.21 0.52		0.67
Glb				0.89
Jchain	P01592	0.19		
Igkc	P01837	0.40		
Ighg	P01863	0.32		
Alb	P07724	0.61		0.83

## Discussion

In the present study, the syndrome and mechanism of thyrotoxicosis mice were studied by routine detection combined with proteomics and metabolomics. Blood routine examination showed that excessive thyroxine led to a decrease in the total number of leukocytes, mainly neutrophils, and lymphocytes. It also led to anemia and thrombocytopenia. The flow cytometry test showed a decrease in CD4/CD8 ratio and the number of CD4+IFN-γ+ T cells in thyrotoxicosis mice, which indicated that immunosenescence occurred. Proteomics analysis also showed a decline in immune function, manifested by the downregulation of complement and coagulation cascades. The above evidence indicated that the immune function of thyrotoxicosis mice declined seriously. The proteomics, biochemical examination, and metabonomics analysis together give the reason for immunosenescence, the decrease of functional proteins and irregular metabolites jointly resulted in the consequence, including the downregulated functional proteins, decreased cholesterol, and cholesterol metabolites, and elevated lipid and lipid metabolites.

Although there is a lack of information on the prevalence and determinants of thyroid function and COVID-19, the British Thyroid Association and the Society for Endocrinology issued a statement emphasizing that patients with thyroid diseases (especially thyrotoxicosis) may have a higher risk of complications, and the American Thyroid Association also recommended that patients with thyroid diseases maintain social distance and limit their exposure to COVID-19 (28). Autoimmune factors mask the relationship between thyroxine and immunity in humans, so the relationship between hyperthyroidism and COVID-19 appears very complex (29). The present study suggested that thyrotoxicosis related immunosenescence might be the cause of aggravation of infection disease, including COVID-19, which also explains the susceptibility and severity of the elderly to respiratory infections. The proteomic analysis further provided evidence and mechanism for declined immune function.

The downregulated DEPs in complement and coagulation cascades were highlighted in the proteomics analysis result, which was a sign of a decrease in defense ability. The complement system is the main component of the immune system (30). In the present study, a series of complement components were downregulated, including C1qa, C1qb, C1qc, C1ra, C1sa, C2, C3, C4b, C4bpa, C5, C8a, C8b, C8g, C9, Cfb, Cfd, Cfh, Cfi, Cfp, Masp1 and Mbl1, and a large number of immunoglobulins were downregulated, indicating the decline of humoral immune function in thyrotoxicosis mice, such as resistance to *Staphylococcus aureus* infection. The DEPs in the cluster of blood coagulation were also downregulated, such as F10, F13b, F2, F3, F5, F7, Fga, Fn1, Gp1ba, Klkb1, Kng1, Plg, Serpinf2, Proc, Thbs1, Vwf, Hgfac, Pf4, F12, Proz, Treml1, Hrg, Serpina10. They were involved in blood coagulation and hemostasis, but also accounted for body fluid levels and wound healing. Moreover, the coagulation system also plays an important role in innate immunity, which prevents microbial invasion by forming physical barriers (31). Some members themselves have antibacterial effects, according to STRING analysis, F2, Pf4, and Hrg are antimicrobial peptides that participate in antimicrobial humoral immune response. Antimicrobial peptides are becoming increasingly recognized as important effectors of innate immunity, for example, Hrg is implicated in the regulation of various physiological and pathological processes including angiogenesis, coagulation, fibrinolysis, the formation of immune complexes, apoptotic/necrotic and pathogen clearance, cell adhesion and antimicrobial activity (32). Hrg can fight against Candida, Staphylococcus aureus, and Escherichia coli by direct or indirect action (33–35). The blood Hrg level of systemic inflammatory response syndrome (SIRS) patients was about 50% lower than that of healthy controls, indicating the consumption of HRG and the resulting decline in defense, meaning the Hrg level was negatively correlated with mortality in ICU, and Hrg was used as a biomarker of SIRS treatment (36, 37). The present study suggested that the downregulation of Hrg was one reason for declined resistance in patients with hyperthyroidism, as in the elderly, because Hrg was also found to be downregulated in aged humans (38), and Hrg can be used as a marker of thyrotoxicosis simulating aging.

In the present study, multi-technical approaches found evidence of a disturbance of internal environment homeostasis. Biochemical test results showed the TP, Alb, Glb, LDC-C, and HDL-C levels were decreased significantly in thyrotoxicosis mice, while TG was increased significantly, which indicated that the synthesis and metabolism of protein and lipid were disordered. The proteomics results further supported this point, for example, the downregulations of Alb, many immunoglobulins, and a series of lipoproteins. Moreover, proteomic results provide more information about homeostasis, such as transport proteins. Their downregulation led to the disturbance of osmotic pressure, ion homeostasis, vitamin utilization, lipid transport, pH, and so on.

In a clinical setting, Alb, Ttr, and Transferrin (Trf) are indicators that the nutritional status of patients can greatly affect the prognosis and length of stay (39–42). Serum Alb is the main protein of plasma and has a good binding capacity for water, Ca^2+^, Na^+^, K^+^, zinc, fatty acids, hormones, bilirubin, and drugs. Its main function is regulating the colloidal osmotic pressure of blood. Low Alb levels, even within the normal range, are associated with weakness, trace elements, and inflammatory markers in the general population (43). Compared with Alb, prealbumin (Ttr) has a shorter half-life, faster liver synthesis rate, and predictable catabolic rate. Malnutrition, inflammation, stress, and aging reduce serum Ttr level, therefore, it is a more sensitive indicator than Alb (41). Trf is one iron transport protein that is responsible for the transport of iron from sites of absorption and heme degradation to the sites of storage and utilization, and hypotransferrin mice have severe anemia and tissue iron overload (44). Here, the downregulation of Alb, Ttr, and Trf indicated that there was obvious malnutrition in thyrotoxicosis mice. The results of fecal metabolomics showed that hypoproteinemia was partly attributed to the loss, for amino acids and dipeptides accounted for 58.6% of all up-regulated metabolites in feces. It indicated that intestinal absorption function was declined in thyrotoxicosis mice, which was similar to elderly humans (45). According to current research results, Alb and Ttr can be used as markers of thyrotoxicosis, and their downregulations were the evidence of thyrotoxicosis simulating aging. In clinical, Alb and Ttr were also the markers of disease severity and prognosis for COVID-19 (46, 47), which indicated good Alb or Ttr reserve might be important for combating COVID-19.

In addition to their role in nutrition, Alb and Trf also play an important role in immunity. One recent study showed that Alb could enhance innate immunity by activating the voltage-gated proton channel hHv1 in neutrophils, suggesting that Alb is a congenital immune regulatory factor (48). Trf participated in the resistance of organisms to foreign microorganisms. Human Trf could inhibit the growth of gram-positive (*Staphylococcus aureus*), gram-negative (*Acinetobacter baumannii*), and fungal (*Candida albicans*) pathogens by isolating iron and destroying membrane potential, Trf is a promising new antibacterial drug (49). In addition, copper and selenium homeostasis also played important role in innate immunity, and their transport proteins Ceruloplasmin (CP) and selenoprotein P (Sepp1) were downregulated in thyrotoxicosis mice. CP accounts for regulating the internal environmental balance of copper and iron, it plays an important role in the immune system (50). Selenium can promote T cell proliferation, NK cell activity, and innate immune cell function (51). Sepp1 produced by hepatocytes is the core of selenium homeostasis in the body because it promotes selenium retention and affects the distribution of selenium from the liver to extrahepatic tissues, especially under the condition of selenium deficiency (52). Above all, Alb, Trf, CP, and Sepp1 played important role in maintaining ion homeostasis and innate immunity, their downregulation seriously affected the defense ability of thyrotoxicosis mice. These transport proteins are evidence that nutritional status is closely related to immunity. The correlation analysis also showed that Alb was positively correlated with the immunity factors in complements and coagulation cascades.

The downregulation DEPs affected the homeostasis of ions, and also affected the utilization of vitamins. In the present study, the abundance of Biotinidase (Btd), Transcobalamin II (Tcn2), vitamin D binding protein (VDBP, Gc), Afamin (Afm), Vitamin K-dependent protein C (Proc), and Vitamin K-dependent protein Z (Proz) were downregulated in thyrotoxicosis mice. Vitamins and their related function proteins are essential for maintaining the normal physiological function and the growth of all organisms. For example, Btd gene mutation leads to biotinidase deficiency and biotin absorption and transport disorder in the small intestinal mucosa. Due to the lack of biotin, the function of biotin dependent carboxylase is weakened, and the metabolism of a variety of amino acids, fatty acids, and sugar is abnormal, resulting in the damage to nervous, skin, immune, and other systems (53). Vitamin B12 (VB12) has an important role in cellular metabolism, especially in DNA synthesis, methylation, and mitochondrial metabolism. VB12 deficiency affects individuals of all ages, but most particularly elderly individuals. Tcn2 is responsible for the endocytosis of VB12 from blood to cells which has an important role in the absorption of VB12. Tcn2 deficiency led to intracellular VB12 deficiency which contributed to nervous system deterioration and megaloblastic anemia (54). Vitamin D (VD) is an important part of the endocrine system that controls calcium homeostasis and bone mineralization (55). VDBP is the main carrier of VD, which affects the utilization of VD and its metabolites (56). Low serum VDBP level is not only associated with low bone mineral density (osteopenia and osteoporosis) in postmenopausal women but also related to infertility, endometriosis, polycystic ovary syndrome, spontaneous abortion, and adverse pregnancy outcomes (57, 58). VKDPs are a group of proteins that need vitamin K to conduct carboxylation, including the coagulation factors II, VII, IX, and X, and the anti-coagulation proteins C, S, and Z. Vitamin K and VKDPs were also associated with age-related diseases, such as cardiovascular disease, osteoarthritis, dementia, cognitive impairment, mobility problems and weakness (59). Further, VK and VD played a synergistic role in bone and cardiovascular health (60). Glycoprotein Afm is the fourth member of the albumin gene family. It is mainly expressed in the liver and secreted into the blood. Plasma Afm not only binds and transports VE but also has neuroprotective and bone remodeling activity (61). Although Afm was found to be associated with metabolic syndrome and obesity, in this study, it might be a marker of liver synthetic function and nutritional status, just like Alb (62). In the present study, the downregulation of Tcn2, Btd, Proc, Proz, Gc, and Afm indicated the usage of vitamins might be inefficient in thyrotoxicosis mice, which is not only related to hyperthyroidism related complications but also aging related diseases.

Lipid transport proteins were also downregulated, including lecithin cholesterol acyltransferase (Lcat), Apoa1, Apoa2, Apoa4, Apob, Apoc2, Apod, Apoe, Apoh, Apom, and Clu (Apoj). They participated in lipoprotein particle assembly and the transport of cholesterol, lecithin, lipid, fatty acid, sphingolipid, and vitamins. Their downregulation indicated the disruption of lipid homeostasis and defense ability. In the clinic, malnourished people with changed HDL proteome were vulnerable to early chronic diseases (63).

The Inter α-trypsin inhibitor (ITI) proteoglycan family is a protein glycosaminoglycan protein complex. They are abundant plasma proteins with naturally immunomodulatory effects in most tissues, which play a key role in homeostasis by crosslinking with the hyaluronan (HA) matrix (64, 65). ITIs could inhibit inflammation and sepsis by countering infectious and danger-associated molecular patterns in plasma and tissue (66). In the present study, Itih1, Itih2, Itih3, and Itih4 were downregulated in thyrotoxicosis mice, which also reflected the decline of defense and protective capability. In addition, ITIs have a strong regulatory effect on ovulation, the main physiological reproductive process, and the fertility of female mice with gene knockout has decreased seriously (67). More than that, according to String analysis, Mug1, Pzp, Cst3, Ctsb, and Mst1 were involved in female pregnancy (**Figure 4**), and their downregulation might be the cause of infertility and poor reproductive outcomes in female patients with hyperthyroidism (68).

Acid-base homeostasis is an important sign of internal environment homeostasis. The normal function of almost all physiological processes in the human body depends on maintaining an appropriate acid-base balance (69). Carbonic anhydrase (Ca) contributes to the balance of CO_2_/HCO_3-_ pools dissolved in the blood, maintain the homeostasis of pH values, and facilitate the transport of CO_2_ from tissue to the lung (70). Mice deficient in carbonic anhydrase 2 (Car2−/− mice) have metabolic acidosis, impaired urine acidification, and are deficient in normal intercalated cells (71). The present study showed that the abundance of Ca1 and Ca2 in thyrotoxicosis mice were both downregulated, which might be the reason for hyperthyroidism associated renal tubular acidosis (72). A study showed that the expression of the Ca2 gene in zebrafish decreased with age, and Ca2 can be used as a biomarker of aging (73).

In summary, the functional proteins were very important for the transport and utilization of nutrients, their downregulation might be the cause of high incidence of complications in patients with thyrotoxicosis or hyperthyroidism and for the elderly. Moreover, according to the analysis by STRING online (**Figure 5**), many downregulated DEPs were involved in the cluster of multicellular organism development. It indicated that organ regeneration and repair ability declined, another feature of aging (74). Although excessive thyroxine resulted in the above consequences, the changes of metabolites induced by excessive thyroxine might be the direct cause. Biochemical test and metabonomics analysis displayed the changes of metabolites, especially the decrease of cholesterol metabolites and elevated lipid metabolites, along with the downregulation of lipoproteins, contributed to the lipid metabolism disturbance in thyrotoxicosis mice.

Cholesterol is the basic component of the cell membrane, its biosynthetic and regulatory pathways are ubiquitous in various cells, including immune cells, which play an important regulatory role in innate and adaptive immune activities (75). Although high cholesterol has been advertised as adverse to health, especially in atherosclerosis, studies have shown that low cholesterol is also harmful. Plasma cholesterol is a negative acute phase reactant, total cholesterol is decreased after surgery and under various pathological conditions, such as trauma, sepsis, burn, and liver dysfunction, which is associated with in-hospital mortality (76, 77). The reason may be that cholesterol and apolipoprotein play an important role in pathogen toxin clearance and regulation of inflammatory response (78). Biochemical test results showed the TC, LDC-C, and HDL-C levels were decreased significantly in thyrotoxicosis mice which also contributed to the reduction of defense capability. Although the decrease in serum cholesterol level is a known finding in hyperthyroidism (79), the reason is still unknown. The metabolomics results in the present study might provide an answer.

Cholesterol homeostasis is regulated by the interaction between endogenous cholesterol synthesis, intestinal diet and bile cholesterol absorption, and bile acid synthesis and excretion. Various plasma markers reflect endogenous cholesterol synthesis (lathosterol, desmosterol, mevalonate, squalene), intestinal cholesterol absorption (sitosterol, campesterol, cholestanol), or bile acid synthesis (7α-hydroxy-4-cholesten-3-one (C4)) in healthy people and patients (80). Lanosterol is an intermediate product of cholesterol synthesis (81). In the present study, the endogenous cholesterol synthesis, intestinal cholesterol absorption, bile cholesterol absorption, and bile acid synthesis and excretion were all decreased. Evidence indicated the downregulation of Lanosterol, Campesterol, and bile acids. In addition to Cholesterol, Dihydrocholesterol, and 20a,22b-Dihydroxycholesterol were also found downregulated. The present study proved that a decreased level of cholesterol is attributed to a decrease in synthesis and absorption. The decreased level of bile acids in the present study suggested that the decreased level of cholesterol was not due to the increasing conversion of cholesterol to bile acids, which was in accordance with a previous study (79).

Bile acids are cholesterol derived metabolites, which play a recognized role in the digestion and absorption of dietary fat (82). Bile acids are physiological factors required for nutrient absorption, distribution, metabolism, and excretion. They are also nutrient sensors and metabolic regulators (83). Bile acids also have important immunomodulatory effects (84). The primary bile acids produced by the liver are metabolized into secondary bile acids under the action of intestinal microbes, they play a role in maintaining the intestinal barrier and preventing intestinal pathogens from colonization. These primary and secondary bile acids play a beneficial role in maintaining innate immunity by acting on their receptors at the interface of the host immune system (85). Taurochenodeoxycholic acid has been found to enhance immunity by increasing the CD4+/CD8+ value in peripheral blood in mice (86). Chenodeoxycholic acid can inhibit the lipotoxicity of cardiomyopathy (87). A previous study showed that T3 dose dependently decreased the formation of cholic acid and chenodeoxycholic acid by inhibiting the expression of CYP7A1 and Cyp8b1 human liver cell lines(88).In the present study, more bile acids were found downregulated, including 3a,7a-Dihydroxy-5b-cholestan-26-al, Chenodeoxycholic acid, Alpha-Muricholic acid, Deoxycholic acid, Taurochenodesoxycholic acid, Dihomodeoxycholic acid, and Lithocholic acid. In particular, Chenodeoxycholic acid and Deoxycholic acid decreased to undetectable levels. The downregulation of these bile acids seriously affected the metabolism of the lipids and drugs as well as immunity (89).

Vitamin D (VD) is one product of cholesterol metabolism. In addition to the classical effects related to mineral homeostasis, VD plays a new role in cell proliferation and differentiation, regulation of the innate and adaptive immune system, prevention of cardiovascular and neurodegenerative diseases, and even anti-aging (90). A recent study found that the serum 25-OHVit D levels in hyperthyroidism patients with hypercalcemia were lower than the normal range, and VD3 adjuvant therapy can improve thyroid related antibody levels, thyroid function, and bone metabolism in patients with hyperthyroidism complicated with hypercalcemia (91). An early study showed that aging reduced the ability of the skin to produce previtamin D3 by more than two times (92). Many aging related diseases are associated with decreased VD3 levels, and VD deficiency remains a global public health problem (93). In present study, (23E)-26,26,26,27,27,27-hexafluoro-1alpha,25-dihydroxy-23,24-didehydrovitamin D3 and (22E)-26,26,26,27,27,27-hexafluoro-1alpha,25-dihydroxy-22,23-didehydrovitamin D3 were downregulated in thyrotoxicosis mice. As reported, the potency of 26,26,26,27,27,27-hexafluoro-1 alpha,25-dihydroxyvitamin D3 (26,27-F6-1,25(OH)2D3) to enhance bone calcium (Ca) mobilization was higher than that of 1 alpha,25-dihydroxyvitamin D3 (94), its activity is about 10 times that of 1,25-dihydroxyvitamin D3 (1,25(OH)2D3) (95). It was the first to detect the decrease of 26,27-F6-1,25(OH)2D3 in thyrotoxicosis mice, and which suggested that the 26,27-F6-1,25(OH)2D3 might play an important role in protecting the body from toxicity caused by excessive thyroid. Strategies that can improve VD3 levels, especially 26,27-F6-1,25(OH)2D3 may have a protective effect on thyrotoxicosis or hyperthyroidism patients and the elderly.

Cholesterol can also convert into sex hormones. Sex hormones are not only related to gender development and sexual fertility, but also affect innate and adaptive immunity, and their levels were changed significantly with age (20). Estradiol and Progesterone are physiological partners which maintain the normal menstrual cycle and bone mass (96). The landmark event of female aging is menopause. The postmenopausal state of women is characterized by low circulating levels of estrogen and progesterone, accompanied by muscle loss (97). In the present study, two pregnane steroids were downregulated, they are Progesterone and 17a-Hydroxypregnenolone. The decrease of sex hormones may be the cause of menstrual cycle disorder in hyperthyroidism patients. The most common manifestations in hyperthyroidism patients are simple oligomenorrhea (reduced menstrual volume) and anovulation cycle, which was similar to menopause (98). Moreover, the symptoms of hyperthyroidism are very similar to those of menopause, such as facial flushing, and sweating (99), which might be attributed to the decline of sex hormones. This was another example of thyrotoxicosis mimicking aging.

The decreased level of bile acids proved that the decreased level of Cholesterol was not due to the increasing conversion of cholesterol to bile acids, VD, or sex hormones. Alternatively, it might be due to the increasing conversion to glucocorticoids (GCs). Conversely to the downregulation of bile acids, other cholesterol metabolites GCs were upregulated in thyrotoxicosis mice. They were all Hydroxysteroids, including Tetrahydrocorticosterone, 11b,17a,21-Trihydroxypreg-nenolone, Cortolone, 3a,21-Dihydroxy-5b-pregnane-11,20-dione, 18-Hydroxycorticosterone, Cortisol, 11-Dehydrocorticosterone, Dihydrocortisol, Corticosterone, and Cortol. Their upregulation indicated the increase of endogenic glucocorticoids (eGCs) in plasma, which was in line with a previous study (100). As reported, increased hypothalamic-pituitary-adrenal (HPA) axis activity is associated with Cushing’s syndrome, hyperthyroidism, and aging (101). GC is the most common cause of secondary osteoporosis and the main cause of non-traumatic osteonecrosis (102). It is well known that GCs have an immunosuppressive effect, which inhibits phagocytosis of macrophages and causes lymphocytic lysis and helper T cell (Th) reduction (103). CD4 + T cells were highly sensitive to GC induced apoptosis (104). As reported, eGCs levels increase with age and eGCs can accelerate aging processes in vertebrate species (105, 106). Research also indicated that stress-induced GCs might play a causal role in aging and age-related disorders (107). Aging and chronic stress together lead to abnormal HPA axis activation and the increase of peripheral GC level, accelerating cell aging and premature immunosenescence, including the reduction of primitive T cells, poor immune response to neoantigens, cellular immunity decline, and thymus degeneration, which increases the incidence of immunosenescence related to diseases, such as tumors and COVID-19 (108, 109). The increase of GCs is both the result and the cause of aging. In the present study, the upregulated eGCs in thyrotoxicosis mice were not only the reason for the decline of immunity but also the evidence that the thyrotoxicosis model simulated aging.

Contrary to low cholesterol, the lipids DEMs were upregulated, such as TGs, Fatty acids, and Sphingolipids, which indicated that lipotoxicity occurred in thyrotoxicosis mice. It is generally believed that the increase in blood lipids is due to the use of antithyroid drugs (110). However, the present study proved that excessive thyroxine itself also leads to an increase in TGs levels in serum. Combined with the upregulation of Monoradylglycerols (MGs), Diradylglycerols (DGs), and a large number of upregulated Fatty acids (FAs), a complete map of TGs metabolism in the thyrotoxicosis model was displayed, and the decomposition of adipose tissue caused by thyroxine leads to the increase of FAs, MGs, and DGs, increasing TGs synthesis. At the same time, due to the decrease of lipoproteins, TGs cannot be transported in time, increasing serum TGs levels (111).

TGs and related metabolites were highly correlated with aging and age-related physiological dysfunction, lower ceramide concentration delayed or improved the symptoms of aging human aging (112). Various intermediates in FA metabolism have been shown to cause cell stress and toxicity (lipotoxicity) in adipocytes and other related cell types (including cardiomyocytes, hepatocytes, and immune cells), including Sphingolipids, Ceramides, and DG (113), and they were all upregulated in the serum of thyrotoxicosis mice. Cells treated with sphingosine could rapidly induce mitochondrial membrane potential loss, mitochondria release cytochrome c, and apoptotic cell death (114). Sphingolipids were implicated in the pathophysiology of cardiovascular disease, and inhibiting sphingolipid synthesis could attenuate cardiomyopathic symptoms (115). Sphingolipids are also markers of aging and their upregulation provides more evidence that thyrotoxicosis mimicked aging.

Plasma long‐chain free FAs were found to be inversely correlated with longevity (116). Moreover, long-chain FAs also affect immune cells, for example, a ω-6 polyunsaturated fatty acids (PUFAs) Linoleic acid (18:2) (LA) could destroy mitochondrial function. LA caused more oxidative damage than other free FAs (such as Palmitic acid), which mediated the selective loss (death) of CD4+ T lymphocytes in the liver, accelerating the occurrence of cancer (117). LA decreased the function of immune cells by inhibiting IFN-γ production in CD4+ T cells (118). The severity of ICU patients with COVID-19 was associated with high levels of free fatty acid (119). And lymphopenia (72.3%) and hypoproteinemia (71.6%) were observed in most patients with COVID-19 (120). Administration of LA to mice could simulate the symptoms of COVID-19, including leukopenia, lymphopenia, lymphocyte damage, relative thrombocytopenia, hypercytokinemia, elevated ALT, hypoalbuminemia, hypocalcemia, shock, and renal failure (121). In the present study, LA was also upregulated, which might be the cause of hypoalbuminemia, leukopenia, lymphocyte damage, and loss and dysfunction of CD4 T cells in thyrotoxicosis mice.

On the contrary, ω- 3 FAs have potential use in COVID-19 treatment because they have antioxidant and anti-inflammatory effects, as well as the ability to regulate platelet homeostasis and the risk of thrombosis (122). A pilot study in 100 patients suggested that ω- 3 FAs tended to reduce the incidence rate and mortality of COVID-19 infection (123). As well known, ω-3 FAs had beneficial health effects on many biological processes, such as improving immune status, protecting against infection and allergies, enhancing cognitive ability, optimizing neuromuscular function, and reducing muscle loss (124, 125). ω-3 FAs can regulate lipid metabolism, promote fatty acid oxidation and inhibit fat production, and lead to good lipid distribution and adipocyte metabolism (126). ω-3 FAs improved body composition by reducing cortisol levels (127), suggesting that ω-3 FAs could resist the adverse effects of cortisol. Coincidentally, in thyrotoxicosis mice, the level of cortisol was upregulated, while the ω-3 FAs and their metabolites were downregulated, the less ω-3 FAs resulted in the weakening resistance to the harmful effects of eGCs. The downregulated ω-3 FAs included Docosapentaenoic acid (22n-3) (DPA), Maresin 1, and 17,18‐epoxyeicosatetraenoic acid (17,18‐EpETE). The effects of ω- 3 FAs eicosapentaenoic acid (EPA) and docosahexaenoic acid (DHA) have been widely studied. DPA is the metabolic intermediate of EPA and DHA. Less is known about DPA; however, the available evidence suggests that DPA is superior to EPA and DHA in the terms of health benefits (128). DPA had anti-inflammatory effects and could improve cardiovascular and metabolic diseases, plasma DPA level was inversely associated with total mortality in older people (129). In addition, DPA was particularly beneficial to the neuroprotection and early life development of the elderly (130). DPA is also a precursor of docosanoids, such as Maresin 1, which was also downregulated in thyrotoxicosis mice. Maresin 1 played an important role in organ protection and remission of acute inflammation by enhancing the immunoresolvent functions of macrophages, it was beneficial to maintaining host defense ability, homeostasis, and wound healing (131). 17,18‐EpETE is a lipid metabolite endogenously generated from EPA which exhibited anti‐allergic and anti‐inflammatory properties (132). The downregulation of DPA, Maresin 1, and 17,18‐EpETE indicated the loss of protection and defense ability in thyrotoxicosis mice.

**Figure 10:**
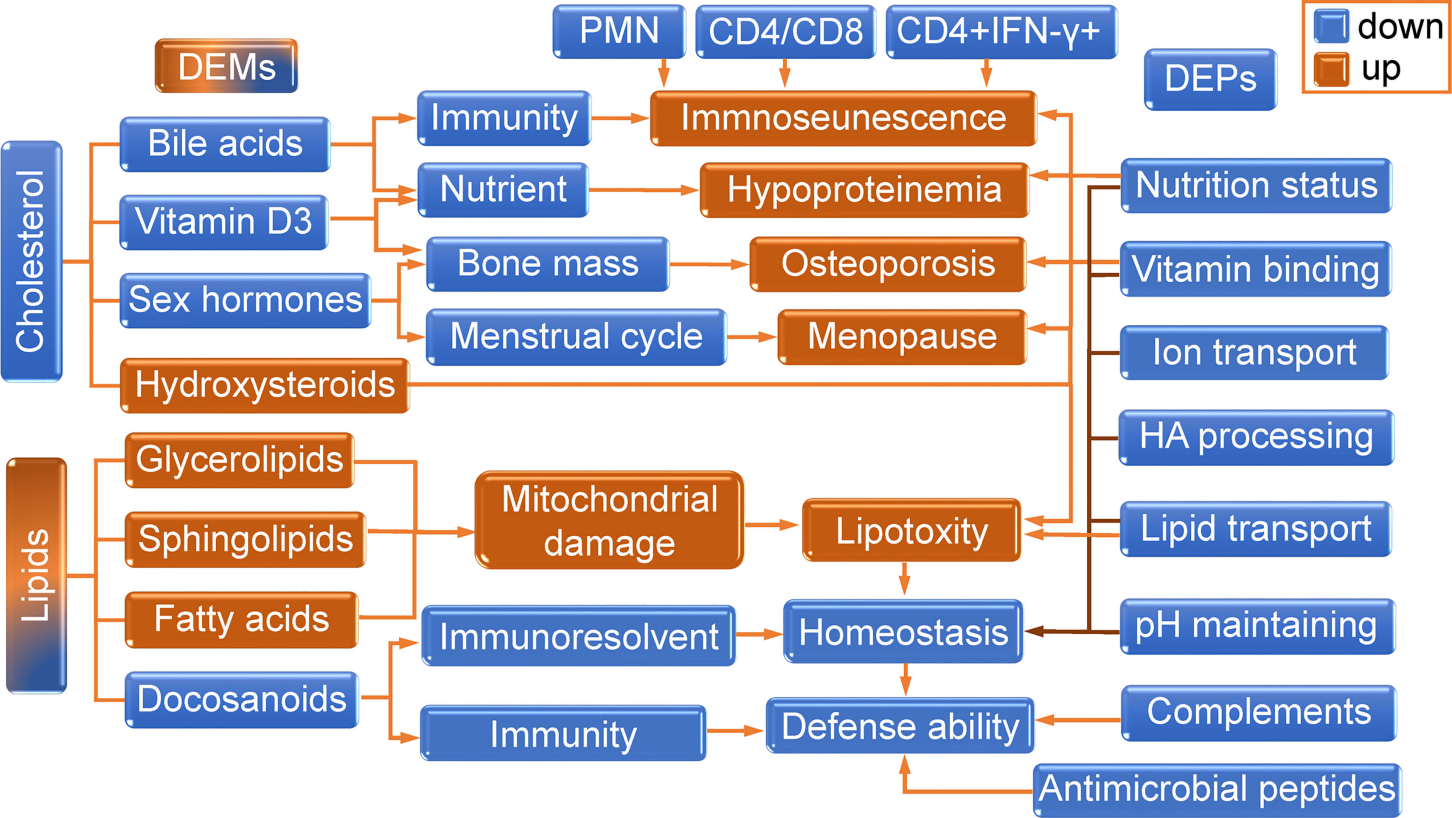
The role of DEPs and DEMs in the aging features of thyrotoxicosis mice. The changes in cholesterol synthesis and metabolism were mainly manifested by the decrease of synthesis and absorption, as well as the increasing conversion to GCs. The decrease of cholesterol, bile acids, pregnane steroids, and VD, as well as excessive GCs, resulted in immunosenescence, osteoporosis, hypoproteinemia, and many other aging related syndromes and diseases. The upregulations of Glycerolipids, Sphingolipids, Fatty Acids were the direct evidence of lipotoxicity. Lipotoxicity is mainly manifested in its damage to the mitochondrial membrane, acting on immune cells leading to immunosenescence, and acting on organs causing organ senescence. GCs are also lipid-like molecules, so the harmful effects of excessive GCs could also be classified as lipotoxicity. On the contrary, the Docosanoids were downregulated, but correspondingly, their decrease also led to the decline of homeostasis regulation ability and defense ability. Downregulated DEPs led to the reduction of lipids, ions and vitamins transmission and utilization efficiency, resulting in malnutrition, which was the main cause of poor prognosis in the elderly. Downregulated DEPs also contributed to the decline of HA processing and PH maintenance. So, changes in metabolites and proteins together destroyed the balance of homeostasis, which consequently led to the low defense response ability and aging related diseases. The decrease of CD4+/CD8+ ratio and IFN-γ production capacity, together with granulocytopenia represented immunosenescence, and the decrease of complements and antimicrobial peptides also represented the declined immune function, all these performances indicated that the defense ability of thyrotoxicosis mice were declined seriously, which were the main causes of susceptibility to infection in the elderly. In conclusion, the present study proved that malnutrition, immunosenescence, and lipotoxicity were the features and mechanisms of thyrotoxicosis mice simulating the accelerated aging model.

The changes in cholesterols and lipotoxicity related DEMs were integrated into **Table 3**. The DEPs and DEMs in the present study worked together to contribute to the aging features of thyrotoxicosis mice, their changes and related effects were integrated into **Figure 10**. In general, changes in cholesterol synthesis and metabolism were mainly manifested by the decreasing synthesis and absorption, as well as the increasing conversion to GCs. The decrease of cholesterol, bile acids, pregnane steroids, and VD, as well as excessive GCs resulted in immunosenescence, osteoporosis, hypoproteinemia, and many other aging related syndromes and diseases. The upregulation of Glycerolipids, Sphingolipids, and FAs was direct evidence of lipotoxicity. Lipotoxicity is mainly manifested in its damage to the mitochondrial membrane, acting on immune cells leading to immunosenescence, acting on organs causing organ senescence. GCs are also lipid-like molecules, so, the harmful effects of excessive GCs could also be classified as lipotoxicity. On the contrary, the Docosanoids were downregulated, but correspondingly, their decrease also led to the decline of defense ability. The downregulation of DEPs resulted in insufficient transportation and utilization of lipids, ions, and vitamins, leading to malnutrition, which is the main cause of poor prognosis in the elderly. Downregulated DEPs also contributed to the decline of HA processing and PH maintenance. Changes in metabolites and proteins together destroyed the balance of homeostasis, which consequently led to the low defense response ability and aging related diseases. The decrease of CD4+/CD8+ ratio and IFN-γ production capacity, together with granulocytopenia represented immunosenescence, and the decrease of complements and antimicrobial peptides represented the declined immune function, all these performances indicated that the defense ability of thyrotoxicosis mice declined seriously, which were the main causes of susceptibility to infection in the elderly. In conclusion, the present study proved that malnutrition, immunosenescence, and lipotoxicity were the mechanisms of thyrotoxicosis mice as well as the features of simulating accelerated aging.

**Table 3:** The changes of cholesterol and lipotoxicity related metabolites.

Metabolites	Compound ID	Formula	FC		P-value	
**Cholesterol synthesis**						
Lanosterol	HMDB0001251	C30H50O	0.69		0.0019	
Campesterol	HMDB0002869	C28H48O	0.34		2.9E-07	
Dihydrocholesterol	HMDB0001569	C27H48O	0.21		0.0001	
20a,22b-Dihydroxycholesterol	HMDB0006763	C27H46O3	0.31		0.0087	
Cholesterol	HMDB0000067	C27H46O	0.70		0.0122	
**Bile acids and derivatives**						
Chenodeoxycholic acid	HMDB0000518	C24H40O4	1.2E-08		3.6E-06	
Chenodeoxycholic acid 3-glucuronide	LMST05010022	C30H48O10	0.87		0.0091	
Deoxycholic acid	HMDB0000626	C24H40O4	7.3E-08		0.0006	
3a,7a-Dihydroxy-5b-cholestan-26-al	HMDB0006894	C27H46O3	0.18		0.0005	
Taurochenodesoxycholic acid	HMDB0000951	C26H45NO6S	0.006		0.0008	
Alpha-Muricholic acid	HMDB0000506	C24H40O5	0.05		0.0027	
Dihomodeoxycholic acid	LMST04020031	C26H44O4	0.48		0.0123	
Lithocholic acid	LMST04010003	C24H40O3	0.69		0.0136	
**Vitamin D**						
25-hydroxyvitamin D2 25-(beta-glucuronide)	LMST05010021	C34H52O8	0.84		0.0462	
(23E)-26,26,26,27,27,27-hexafluoro-1alpha,25-dihydroxy-23,24-didehydrovitamin D3	LMST03020083	C27H36F6O3	0.42		0.0021	
(22E)-26,26,26,27,27,27-hexafluoro-1alpha,25-dihydroxy-22,23-didehydrovitamin D3	LMST03020082	C27H36F6O3	0.44		0.0058	
**Hydroxysteroids**						
Tetrahydrocorticosterone	HMDB0000268	C21H34O4	2.28		0.0318	
11b,17a,21-Trihydroxypreg-nenolone	HMDB0006760	C21H32O5	2.46		0.0446	
Cortolone	HMDB0003128	C21H34O5	3.67		0.0052	
3a,21-Dihydroxy-5b-pregnane-11,20-dione	HMDB0006755	C21H32O4	3.90		0.0042	
18-Hydroxycorticosterone	HMDB0000319	C21H30O5	6.55		0.0263	
Cortisol	HMDB0000063	C21H30O5	14.26		9.8E-07	
11-Dehydrocorticosterone	HMDB0004029	C21H28O4	14.68		7.6E-06	
Dihydrocortisol	HMDB0003259	C21H32O5	146.11		0.0024	
Corticosterone	HMDB0001547	C21H30O4	209.90		0.0335	
Cortol	HMDB0003180	C21H36O5	382.37		0.0003	
11-Dehydrocorticosterone	LMST02030192	C21H28O4	1.86		0.0245	
Corticosterone	LMST02030186	C21H30O4	2.56		0.0376	
**Glycerolipids**						
DG(18:2(9Z,12Z)/18:2(9Z,12Z)/0:0)	HMDB0007248	C39H68O5	2.18		0.0411	
DG(16:0/18:2(9Z,12Z)/0:0)	HMDB0007103	C37H68O5	3.85		0.0136	
DG(16:1(9Z)/18:2(9Z,12Z)/0:0)	HMDB0007132	C37H66O5	10.80		0.0211	
DG(18:1(9Z)/18:2(9Z,12Z)/0:0)[iso2]	LMGL02010056	C39H70O5	3.90		0.0009	
1-O-(2R-hydroxy-hexadecyl)-sn-glycerol	LMGL01020063	C19H40O4	1.93		0.0298	
TG(18:3(6Z,9Z,12Z)/20:0/22:6(4Z,7Z,10Z,13Z,16Z,19Z))[iso6]	LMGL03016429	C63H104O6	2.22		0.0210	
TG(12:0/18:3(9Z,12Z,15Z)/19:1(9Z))[iso6]	LMGL03013521	C52H92O6	6.20		0.0006	
TG(13:0/14:0/22:4(7Z,10Z,13Z,16Z))[iso6]	LMGL03013699	C52H92O6	6.74		0.0010	
TG(12:0/17:1(9Z)/20:3(8Z,11Z,14Z))[iso6]	LMGL03013408	C52H92O6	7.78		0.0008	
**Sphingolipids**						
GM4(d18:1/16:0)	LMSP0601AA01	C51H94N2O16	1.48		0.0270	
CerP(d18:1/18:0)	LMSP02050004	C36H72NO6P	1.73		0.0240	
Cer(d18:0/14:0)	LMSP02020016	C32H65NO3	1.79		0.0337	
GlcCer(d15:2(4E,6E)/20:0)	LMSP0501AA59	C41H77NO8	1.51		0.0595	
GlcCer(d15:2(4E,6E)/22:0)	LMSP0501AA60	C43H81NO8	1.82		0.0219	
N-(hexadecanoyl)-deoxysphing-4-enine-1-sulfonate	LMSP00000002	C34H67NO5S	1.70		0.0106	
N-(tetradecanoyl)-deoxysphing-4-enine-1-sulfonate	LMSP00000001	C32H63NO5S	1.98		0.0203	
SM(d16:1/20:0)	LMSP03010052	C41H83N2O6P	1.80		0.0727	
SM(d18:2/24:0)	LMSP03010081	C47H93N2O6P	5.65		0.0109	
SM(d18:1/24:1(15Z))	LMSP03010007	C47H93N2O6P	6.57		0.0109	
LysoSM(d18:1)	HMDB0006482	C23H50N2O5P	2.15		0.0426	
Sphingosine 1-phosphate	HMDB0000277	C18H38NO5P	2.45		0.0003	
Sphinganine 1-phosphate	HMDB0001383	C18H40NO5P	3.69		0.0009	
SM(d18:1/24:1(15Z))	HMDB0012107	C47H93N2O6P	7.29		3E-05	
**Fatty Acids and Conjugates**						
Hydroxyphthioceranic acid (C40)	LMFA01020326	C40H80O3	1.25		0.0256	
6-bromo-tricosa-5E,9Z-dienoic acid	LMFA01090101	C23H41BrO2	1.28		0.0116	
29:2(5Z,9Z)(6Br)	LMFA01030891	C29H53BrO2	1.29		0.0004	
Linoleic acid	LMFA01030120	C18H32O2	1.89		0.0083	
Oleic acid	LMFA01030002	C18H34O2	2.46		0.0077	
10-hydroxy-16-oxo-hexadecanoic acid	LMFA01170060	C16H30O4	1.46		0.0246	
trans-9-palmitoleic acid	LMFA01030057	C16H30O2	2.18		0.0036	
13-hexadecenoic acid	LMFA01030263	C16H30O2	3.01		0.0026	
6Z,9Z-hexadecadienoic acid	LMFA01030273	C16H28O2	3.05		0.0119	
Tetradecanedioic acid	LMFA01170018	C14H26O4	2.90		0.0010	
Tetranor-8-NO2-CLA	LMFA01120009	C14H23NO4	1.4E+08		0.0230	
2-methyl-dodecanedioic acid	LMFA01170010	C13H24O4	1.57		0.0130	
11R-hydroxy-dodecanoic acid	LMFA01050253	C12H24O3	1.38		0.0301	
11-hydroxy-dodecanoic acid	LMFA01050165	C12H24O3	1.39		0.0302	
9-hydroxy-dodecanoic acid	LMFA01050167	C12H24O3	1.43		0.0105	
xi-5-Hydroxydodecanoic acid	LMFA01050529	C12H24O3	1.47		0.0162	
4-hydroxy lauric acid	LMFA01050038	C12H24O3	1.54		0.0048	
3-hydroxy-dodecanedioic acid	LMFA01160025	C12H22O5	1.88		0.0248	
Oleic acid	HMDB0000207	C18H34O2	1.39		0.0027	
Palmitoleic acid	HMDB0003229	C16H30O2	2.81		0.0096	
Palmitelaidic acid	HMDB0012328	C16H30O2	2.10		0.0140	
Beta-hydroxymyristic acid	HMDB0061656	C14H28O3	1.72		0.0363	
**Docosanoids and metabolites**						
DPA	LMFA04000044	C22H34O2	6E-08		0.0448	
Docosapentaenoic acid (22n-3)	HMDB0006528	C22H34O2	6E-08		0.0020	
Maresin 1	LMFA04050001	C22H32O4	0.78		0.0196	
17,18-EpETE	HMDB0010212	C20H30O3	0.12		0.0065	

The present study suggests that the thyrotoxicosis model is an ideal accelerated aging model, which can be used to study the mechanism of aging and can even be used to explore disease prevention measures and drugs, such as those related to COVID-19. The model could also be adapted for exploring drugs and strategies to improve the prognosis of hospitalized elderly patients. Candidates that can improve the symptoms or markers of thyrotoxicosis may have potential value in the development of anti-aging drugs.

## Data Availability Statement

The original contributions presented in the study are publicly available. This data can be found here: https://www.iprox.cn/page/HMV006.html under the accession number IPX0004071000.

## Ethics Statement

The animal study was reviewed and approved by Animal Care and Use Committee of Shandong Province, China.

## Author Contributions

GZ conceived the project and designed the experiments. QF, WX, GD, JL, and JY conducted the experiments. QF and DL performed proteomic analysis and wrote the paper. All authors contributed to the article and approved the submitted version.

## Conflict of Interest

Author QF, WX, GD, JL, JY, and GZ are employed by Lunan Pharmaceutical Group Co., Ltd.

The remaining authors declare that the research was conducted in the absence of any commercial or financial relationships that could be construed as a potential conflict of interest.

## Publisher’s Note

All claims expressed in this article are solely those of the authors and do not necessarily represent those of their affiliated organizations, or those of the publisher, the editors and the reviewers. Any product that may be evaluated in this article, or claim that may be made by its manufacturer, is not guaranteed or endorsed by the publisher.
